# Complete Genome Sequence of *Vibrio coralliilyticus* 58, Isolated from Pacific Oyster (*Crassostrea gigas*) Larvae

**DOI:** 10.1128/genomeA.00437-17

**Published:** 2017-06-08

**Authors:** Hyoun Joong Kim, Ji Hyung Kim, Jin Woo Jun, Sib Sankar Giri, Cheng Chi, Saekil Yun, Sang Guen Kim, Sang Wha Kim, Jeong Woo Kang, Dae Gwin Jeong, Se Chang Park

**Affiliations:** a Laboratory of Aquatic Biomedicine, College of Veterinary Medicine and Research Institute for Veterinary Science, Seoul National University, Seoul, Republic of Korea; b Infectious Disease Research Center, Korea Research Institute of Bioscience and Biotechnology, Daejeon, Republic of Korea

## Abstract

We report here the complete genome of *Vibrio coralliilyticus* strain 58, which was originally isolated from inactive Pacific oyster (*Crassostrea gigas*) larvae in Japan. The assembled genome consisted of two chromosomes and one plasmid. These data will provide valuable information and important insights into the biodiversity of this organism.

## GENOME ANNOUNCEMENT

*Vibrio coralliilyticus* is a well-known pathogen responsible for the drastic losses in coral reefs worldwide (1). Moreover, this bacterium has been shown to infect crustaceans, fish (2), and bivalves, including the Pacific oyster (3, 4). In Japan, bacillary necrosis leading to mass mortalities in Pacific oyster hatcheries has been documented continuously since 1995, with the causative agent being biochemically identified as *V. splendidus* biovar II 58 (5). However, our recent analysis established that these isolates should be reclassified as *V. coralliilyticus*. Here, we present the complete genome of *V. coralliilyticus* 58, isolated from a Pacific oyster in Japan.

Bacterial genomic DNA was isolated using a DNeasy blood and tissue kit (Qiagen) following the manufacturer’s protocols. Sequencing was performed by Macrogen, Inc. on the PacBio RS II system (Pacific Biosciences), following construction of a 20-kb SMRTbell template library. The sequences generated (1,299,427,830 bp; 221,596 reads) were assembled using the Hierarchical Genome Assembly Process version 3.0, and the assembled genome comprised 5,490,011 bp, consisting of two chromosomes named Chr I (3,504,421 bp) and Chr II (1,933,141 bp), and one plasmid designated pVs58 (52,449 bp). Genome annotation was acquired from the NCBI Prokaryotic Genome Annotation Pipeline (http://www.ncbi.nlm.nih.gov/books/NBK174280), which revealed 4,984 genes, 4,832 coding sequences, 101 pseudogenes, 34 rRNAs (5S, 16S, and 23S), 114 tRNAs, and 4 noncoding RNAs.

Overall genome similarity among *V. coralliilyticus* 58 and other *Vibrio* sp. strains was assessed using the orthologous average nucleotide identity (OrthoANI) algorithm (6), and the result indicated >96% genome similarity to other *V. coralliilyticus* strains OCN014 (7) and RE98 (8). To date, the extracellular metalloprotease (*VtpA*) has been verified as one of the critical virulence factors of *V. coralliilyticus* (9), and the corresponding *vtpA* gene has been reported in strain RE22 (10). Similarly, the *vtpA* (91.7% sequence identity) gene homologous to those of *V. coralliilyticus* were found on Chr II of strain 58 in the present study.

Interestingly, the cytolysin/hemolysin gene (94.3% sequence identity), which is homologous to *vthB/A* gene in other *V. coralliilyticus* strains (9), was found to be encoded by pVs58, suggesting that this putative virulence plasmid might also be associated with the pathogenicity of *V. coralliilyticus*. Moreover, a BLAST search showed that pVs58 shared no continuous sequence identity with other plasmids in GenBank and was most similar to pOCN014 (from *V. coralliilyticus* OCN14; 80% identity) and p319 (from *V. coralliilyticus* RE98; 79% identity), with only 20% coverage. These results strongly support our recent reclassification of *V. splendidus* biovar II 58 as *V. coralliilyticus* 58 and demonstrate the uniqueness of this strain compared to the other *V. coralliilyticus* strains sequenced to date.

In conclusion, the complete genome sequence of *V. coralliilyticus* 58 and associated genomic data will provide important insights into the biodiversity of the *Vibrio* genus and valuable information for the study of virulence factors, facilitating control of *V. coralliilyticus* infections in aquaculture.

### Accession number(s).

This whole-genome shotgun project has been deposited in GenBank under the accession numbers CP016556 (Chr I), CP016557 (Chr II), and CP016558 (plasmid pVs58). The versions described in this paper are the first versions, CP016556.1, CP016557.1, and CP016558.1, respectively. The BioProject ID is PRJNA327286.
